# Genomic Identification of Two Phytobacter diazotrophicus Isolates from a Neonatal Intensive Care Unit in Singapore

**DOI:** 10.1128/mra.00167-23

**Published:** 2023-05-11

**Authors:** Peiyun Hon, Karrie K. K. Ko, Jonathan C. W. Zhong, Partha P. De, Theo H. M. Smits, Jiaming Low, Shawn Vasoo, Clement K. M. Tsui

**Affiliations:** a Infectious Diseases Research Laboratory, National Centre for Infectious Diseases, Tan Tock Seng Hospital, Singapore, Singapore; b Department of Microbiology, Singapore General Hospital, Singapore, Singapore; c Genome Institute of Singapore, Agency for Science, Technology and Research, Singapore, Singapore; d Department of Laboratory Medicine, Tan Tock Seng Hospital, Singapore, Singapore; e Environmental Genomics and Systems Biology Research Group, Institute of Natural Resource Sciences (IUNR), Zurich University of Applied Sciences (ZHAW), Wädenswil, Switzerland; f Department of Neonatology, National University Hospital, Singapore, Singapore; g Lee Kong Chian School of Medicine, Nanyang Technological University, Singapore, Singapore; h Department of Infectious Diseases, Tan Tock Seng Hospital, Singapore, Singapore; i Yong Loo Lin School of Medicine, National University of Singapore, Singapore, Singapore; j Faculty of Medicine, University of British Columbia, Vancouver, British Columbia, Canada; Loyola University Chicago

## Abstract

We report the draft genome sequences of two Phytobacter diazotrophicus isolates recovered from a swab specimen from the water faucet located in the Neonatal Intensive Care Unit (ICU), National University Hospital, Singapore. The isolates were misidentified as Cronobacter sakazakii and Klebsiella oxytoca using biochemical methods. Whole-genome sequencing (WGS) was performed to determine their identity.

## ANNOUNCEMENT

Members of the genus *Phytobacter* (order *Enterobacterales*) are isolated from the natural environment and clinical settings (1, 2). They are known as saprobes but increasingly reported in clinical infections (1, 2). Identification of *Phytobacter* strains based on biochemical characteristics is complicated due to taxonomic confusion, and they are often misidentified by automated identification systems in laboratories (1). Here, we report the identification of two Phytobacter diazotrophicus isolates using whole-genome sequencing (WGS) data.

Strains 2A and 2B were isolated from a swab specimen taken from the water faucet (i.e., p-trap and water faucet outlet) located in the milk preparation room of a neonatal ICU in National University Hospital, Singapore. Briefly, the ESwabs (Copan Diagnostics) were placed in the buffer and vortexed for 10 s, and 100 μL of Amies medium was plated on tryptic soy agar (TSA) sheep blood and MacConkey plates, which were incubated overnight at 35 ± 2°C. Colonies were identified using the MALDI Biotyper system based on the Microflex LT mass spectrometer (Bruker, USA) and Microbact kit (Thermo Fisher Scientific, Massachusetts). Antimicrobial susceptibility testing (AST) was performed using Oxoid antimicrobial susceptibility disks (Thermo Fisher). The MICs of antibiotics were interpreted according to the CLSI breakpoints for *Enterobacterales* (3).

Bacteria were cultured on blood agar at 35°C overnight prior to DNA extraction using the MagNA Pure system (Roche, Switzerland). DNA concentrations were measured using the Qubit 4 fluorimeter (Thermo Fisher Scientific), and DNA libraries were constructed using a DNA prep kit and adapters (Illumina, Massachusetts). Sequencing was performed on the Illumina MiSeq platform to generate 300-bp paired-end reads. The reads were quality trimmed using Trim Galore v.0.6.5 (http://www.bioinformatics.babraham.ac.uk/projects/trim_galore/), and the quality was assessed using FastQC v.0.11.9 (https://github.com/s-andrews/FastQC). The reads were assembled using SPAdes v.3.9.0 (4). Small contigs (<500 bp) were discarded. The assembly statistics were assessed using QUAST v.5.0.2 (5). Antimicrobial resistance genes were predicted using ResFinder v.3.2 (6) and PlasmidFinder (7) through ABRicate v.0.9.8 (https://github.com/tseemann/abricate) based on ≥70% coverage and ≥90% sequence identity. The genomes were uploaded to the Type (Strain) Genome Server (TYGS) (https://tygs.dsmz.de) (8) to determine their relationship with other bacteria. FastANI (9) was used to compute the genetic distances. The genomes were annotated using the NCBI Prokaryotic Genome Annotation Pipeline v.4.11 (10). Default parameters were used for all software, unless otherwise specified.

The isolates were determined to be *Cronobacter* sp. (1.86) and Klebsiella oxytoca (1.84), respectively (with low confidence scores), using matrix-assisted laser desorption ionization–time of flight (MALDI-TOF) mass spectrometry and Cronobacter sakazakii (93.6%) using Microbact. Owing to the conflicting results from the biochemical methods, WGS data were utilized to resolve the confusion. The isolates were identified as most closely related to *P. diazotrophicus* DSM 17806 (GenBank accession number GCA_004346725) (d_0_ = 80.8%) using TYGS, and they shared 99.9% genomic similarity on average; the genomic and phenotypic information is summarized in Table 1. The two strains whose genomes are reported here possess the beta-lactamase genes *bla*_CTX-M-9_ and *bla*_SHV-12_ (2), consistent with the AST report as extended-spectrum beta-lactamase-producing *Enterobacterales* members. Noteworthy, the isolates carried *mcr-9*, a variant of *mcr-1*. A BLAST search of the contig (2,002 bp) containing *mcr-9* in strain 2B against NCBI databases indicated 100% identity to the plasmids of multiple *Enterobacterales* isolates, two of which (CP052871.1 and CP050163.1) were annotated as replicon type IncHI2. The replicon IncHI2 was also present in strains 2A and 2B (Table 1), though not linked to the *mcr-9*-bearing contig. However, the presence of *mcr-9* in 2A and 2B was not associated with resistance to polymyxin B, as in previous reports (11, 12). These isolates also carried the genes *sul1*, *dfrA16*, *catA1*, *ant(2″)-Ia*, and *aadA2*, which are associated with resistance to the antibiotics trimethoprim-sulfamethoxazole, chloramphenicol, gentamicin, and streptomycin. Given that the *P. diazotrophicus* strains were resistant to multiple antibiotics and were misidentified using common diagnostic methods, the role of this species in the healthcare environment and human colonization or infection may have been hitherto underrecognized.

**TABLE 1:** Summary of genome statistics, genetic mechanisms of antibiotic resistance and biochemical identification tests

Characteristic	Data for strain:	
	2A	2B
GenBank accession no.	JAQNCX000000000	JAQNCW000000000
Genome size (bp)	5,935,433	5,936,610
No. of reads	2,318,604	3,665,674
*N*_50_ (bp)	148,979	153,602
GC content (%)	52.65	52.65
Avg coverage (×)	110	180
No. of contigs	135	131
No. of CDS^*a*^	5,707	5,703
Predicted antimicrobial resistance genes (ResFinder)	*bla*_SHV-12_, *bla*_CTX-M-9_, *mcr-9*, *ant(2″)-Ia*, *oqxB*, *oqxA*, *aadA2*, *sul1*, *catA1*, *dfrA16*	*bla*_SHV-12_, *bla*_CTX-M-9_, *mcr-9*, *ant(2″)-Ia*, *oqxB*, *oqxA*, *aadA2*, *sul1*, *catA1*, *dfrA16*
Predicted plasmids	IncHI2, IncHI2A, pKPC-CAV1321, Col440II, Col(pHAD28)	IncHI2, IncHI2A, pKPC-CAV1321, Col440II, Col(pHAD28)
Microbact result (%)		
Closest match	Cronobacter sakazakii (93.57)	Cronobacter sakazakii (93.57)
Second closest match	Enterobacter amnigenus biogp 1 (6.06)	Enterobacter amnigenus biogp 1 (6.06)
MALDI-TOF result (first run [%])		
Closest match	*Cronobacter* sp. (1.86)	Klebsiella oxytoca (1.84)
Second closest match	Klebsiella oxytoca (1.82)	Salmonella sp. (1.8)
MALDI-TOF result (second run [%])		
Closest match	Klebsiella aerogenes (1.84)	*Cronobacter* sp. (1.9)
Second closest match	*Cronobacter* sp. (1.83)	*Cronobacter* sp. (1.89)

a CDS, coding DNA sequences.

### Data availability.

The whole-genome shotgun data from this study have been deposited in the DDBJ/ENA/GenBank repositories under accession numbers JAQNCW010000000 and JAQNCX010000000 and BioProject accession number PRJNA918442. The versions described in this paper are the first versions.
