# Correction for Hon et al., “Genomic Identification of Two *Phytobacter diazotrophicus* Isolates from a Neonatal Intensive Care Unit in Singapore”

**DOI:** 10.1128/MRA.00689-23

**Published:** 2023-09-25

**Authors:** Peiyun Hon, Karrie K. K. Ko, Jonathan W. Z. Chia, Partha P. De, Theo H. M. Smits, Jiaming Low, Shawn Vasoo, Clement K. M. Tsui

## AUTHOR CORRECTION

Volume 12, no. 6, e00167-23, 2023, https://doi.org/10.1128/mra.00167-23. Jonathan W. Z. Chia’s name was rendered incorrectly as “Jonathan C. W. Zhong” in the byline. The byline should appear as shown in this author correction.

